# Crystal structure of 2-hy­droxy-2-phenyl­aceto­phenone oxime

**DOI:** 10.1107/S2056989020016163

**Published:** 2021-01-01

**Authors:** Nurcan Akduran

**Affiliations:** a Department of Metallurgical and Materials Engineering, Faculty of Technology, Selçuk University, 42130 Selçuklu, Konya, Turkey

**Keywords:** crystal structure, hydrogen bond, phenyl­aceto­phenone, oxime

## Abstract

The title compound [systematic name: 2-(*N*-hy­droxy­imino)-1,2-di­phenyl­ethanol], C_14_H_13_NO_2_, consists of hy­droxy phenyl­aceto­phenone and oxime units, in which the phenyl rings are oriented at a dihedral angle of 80.54 (7)°. In the crystal, inter­molecular O—H_Oxm_⋯N_Oxm_, O—H_Hydr_⋯O_Hydr_, O—H′_Hydr_⋯O_Hydr_ and O—H_Oxm_⋯O_Hydr_ hydrogen bonds link the mol­ecules into infinite chains along the *c*-axis direction. π–π contacts between inversion-related of the phenyl ring adjacent to the oxime group have a centroid–centroid separation of 3.904 (3) Å and a weak C—H⋯π(ring) inter­action is also observed. A Hirshfeld surface analysis of the crystal structure indicates that the most important contributions for the crystal packing are from H⋯H (58.4%) and H⋯C/C⋯H (26.4%) contacts. Hydrogen bonding and van der Waals contacts are the dominant inter­actions in the crystal packing.

## Abstract 2

The phenyl rings are almost oriented perpendicularly. In the crystal, inter­molecular O—H_Oxm_⋯N_Oxm_, O—H_Hydr_⋯O_Hydr_, O—H′_Hydr_⋯O_Hydr_ and O—H_Oxm_⋯O_Hydr_ hydrogen bonds link the mol­ecules into infinite chains along the *c*-axis direction.

## Chemical context   

Inter­molecular hydrogen bonding has received considerable attention among the directional non-covalent inter­molecular inter­actions (Etter *et al.*, 1990 ▸). Hydrogen bonds combine moderate strength and directionality (Karle *et al.*, 1996 ▸) in linking mol­ecules to form supra­molecular structures. The oxime (–C=N—OH) moiety, which is similar to carb­oxy­lic acid in that it contains one hydrogen-bond donor and two acceptor atoms, is a functional group that has not been extensively explored in crystal engineering. Structurally characterized oxime moieties are much less common than carb­oxy­lic acids and amides, but from a supra­molecular perspective, this functionality does have some unique and desirable features (Aakeröy *et al.*, 2001 ▸). Oxime groups possess stronger hydrogen-bonding capabilities than alcohols, phenols and carb­oxy­lic acids (Marsman *et al.*, 1999 ▸). The hydrogen-bond systems in the crystals of oximes have been analysed and a correlation between patterns of hydrogen bonding and N—O bond lengths has been suggested (Bertolasi *et al.*, 1982 ▸). Oxime and dioxime derivatives are very important in the chemical industry, photography, agriculture, textiles, technological improvement, dye chemistry, semiconductor manufacturing and medicine (Sevagapandian *et al.*, 2000 ▸; Schrauzer *et al.*, 1965 ▸; Thomas & Underhill, 1972 ▸; Underhill *et al.*, 1973 ▸; Chakravorty, 1974 ▸; Kurita, 1998 ▸; Mathur & Narang, 1990 ▸; Ravi Kumar, 2000 ▸). They have a broad pharmacological activity spectrum, encompassing anti­bacterial, anti­depressant and anti­fungal activities (Forman, 1964 ▸; Holan *et al.*, 1984 ▸). Some oxime complexes also have anti­carcinogenic activities (Sevagapandian *et al.*, 2000 ▸; Srivastava *et al.*, 1997 ▸). The crystallization and the mol­ecular and crystal structures of the title compound, (I), are reported herein. Its magnetic properties have previously been studied by electron paramagnetic resonance (EPR) (Sayin *et al.*, 2012 ▸).
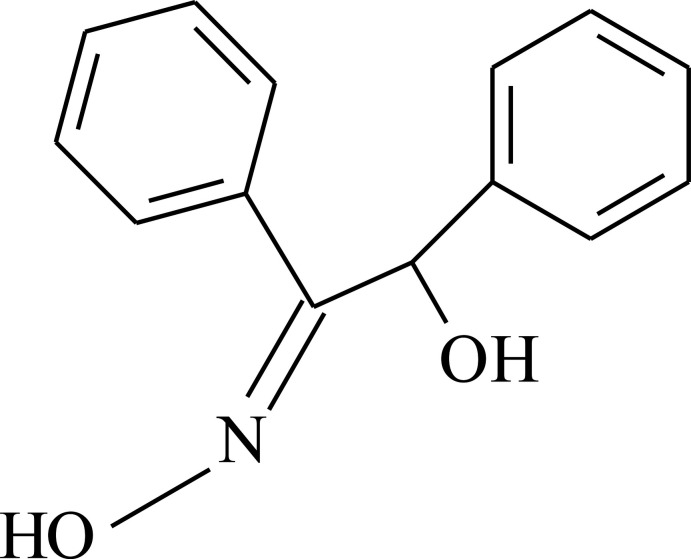


## Structural commentary   

**Figure 1:**
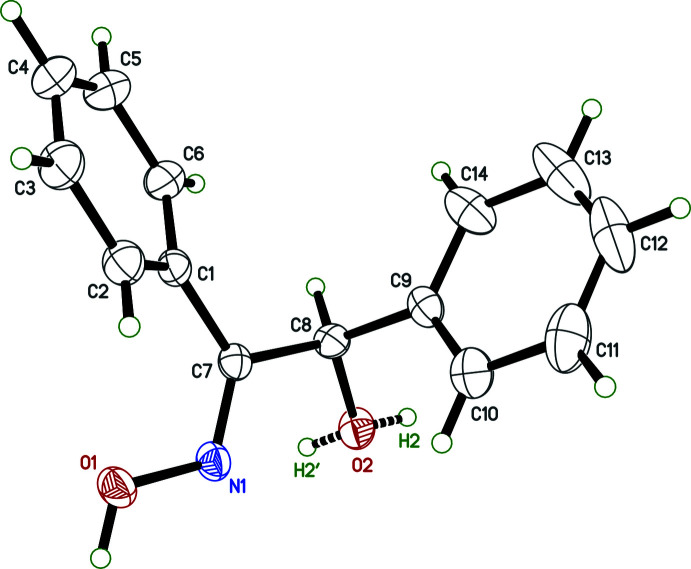
The asymmetric unit of the title compound with the atom-numbering scheme. Displacement ellipsoids are drawn at the 30% probability level. The hydrogen attached to hydroxyl oxygen O2 is disordered in a 50:50 ratio, as indicated by dashed bonds.

As shown in Fig. 1 ▸, the title compound, (I), consists of hy­droxy phenyl­aceto­phenone and oxime units, where the phenyl, *A* (C1–C6) and *B* (C9–C14), rings are oriented at a dihedral angle of 80.54 (7)°. The dihedral angle between the oxime plane *C* (O1/N1/C7) and phenyl rings *A* and *B* are 39.48 (9) and 80.11 (14)°, respectively. The base of the oxime moiety is approximately coplanar with the *A* phenyl ring plane, as indicated by the O1—N1—C7—C1 torsion angle of 1.0 (3)°. In the oxime moiety, the O1—N1 [1.4026 (18) Å] bond length and the O1—N1—C7 [115.36 (14)°] bond angle may be compared with the corresponding values of O1—N2 [1.423 (3) Å], O2—N3 [1.396 (3) Å], O2—N3—C10 [111.5 (2)°] and O1—N2—C9 [109.4 (2)°] in the glyoxime moiety reported in 1-(2,6-di­methyl­phenyl­amino)­propane-1,2-dione dioxime [(II); (Hökelek *et al.*, 2001 ▸)], reflecting the types and electron-withdrawing or donating properties of the substituents bonded to the carbon atoms of the glyoxime moiety.

## Supra­molecular features   

**Figure 2:**
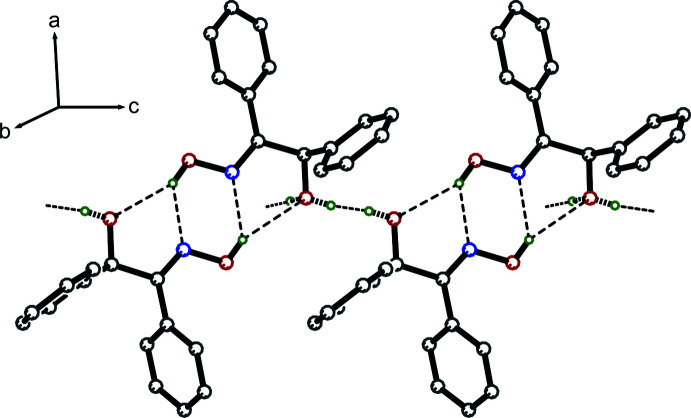
A partial packing diagram. The O—H_Oxm_⋯N_Oxm_, O—H_Hydr_⋯O_Hydr_, O—H′_Hydr_⋯O_Hydr_ and O—H_Oxm_⋯O_Hydr_ hydrogen bonds [Oxm = oxime and Hydr = hy­droxy] are shown as thin dashed lines. Bonds involving the disordered hydroxyl hydrogen are shown as thick dashed lines. The remaining hydrogen atoms have been omitted for the sake of clarity.

In the crystal, inter­molecular O—H_Oxm_⋯O_Hydr_, O—H_Oxm_⋯N_Oxm_, O—H_Hydr_⋯O_Hydr_ and O—H′_Hydr_⋯O_Hydr_ hydrogen bonds (Table 1 ▸, Fig. 2 ▸) [Oxm = oxime and Hydr = hy­droxy] form 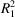
(5) and 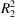
(6) ring motifs (Etter *et al.*, 1990 ▸) between inversion-related mol­ecules, which link to form extended chains along the *c*-axis direction (Figs. 2 ▸ and 3 ▸). π–π contacts between inversion-related phenyl rings [*Cg*1⋯*Cg*1^i^ = 3.904 (1) Å; symmetry code: (i) 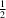 − *x*, 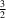 − *y*, 1 − *z*), where *Cg*1 is the centroid of ring *A* (C1–C6)] and a weak C—H⋯π(ring) inter­action (Table 1 ▸) are also observed. A Hirshfeld surface analysis of the crystal structure indicates that the most important contributions for the crystal packing are from H⋯H (58.4%) and H⋯C/C⋯H (26.4%) inter­actions, but a full Hirshfeld surface analysis is complicated by the disorder. Hydrogen bonding and van der Waals inter­actions comprise the dominant contacts in the crystal packing (Table 2 ▸).

## Synthesis and crystallization   

The alpha-benzoinoxime [ABO (C_14_H_13_NO_2_) powder was purchased from Merck, and crystallized by slow evaporation from a concentrated solution in ethanol as colourless crystals at room temperature.

## Refinement   

Experimental details including the crystal data, data collection and refinement are summarized in Table 3 ▸. The hy­droxy H atoms were located in a difference-Fourier map and refined isotropically. The C-bound H atoms were positioned geometrically, with C—H = 0.93 Å (for aromatic H atoms), and constrained to ride on their parent atoms, with *U*_iso_(H) = 1.2*U*_eq_(C). The hydrogen attached to O2 is disordered over two sites (H2 and H2′) in a 0.5:0.5 ratio and was refined with restraints.

## Supplementary Material

Crystal structure: contains datablock(s) I, global. DOI: 10.1107/S2056989020016163/pk2654sup1.cif

Structure factors: contains datablock(s) I. DOI: 10.1107/S2056989020016163/pk2654Isup2.hkl

Supporting information file. DOI: 10.1107/S2056989020016163/pk2654Isup3.cml

CCDC reference: 2049764

Additional supporting information:  crystallographic information; 3D view; checkCIF report

## Figures and Tables

**Figure 3:**
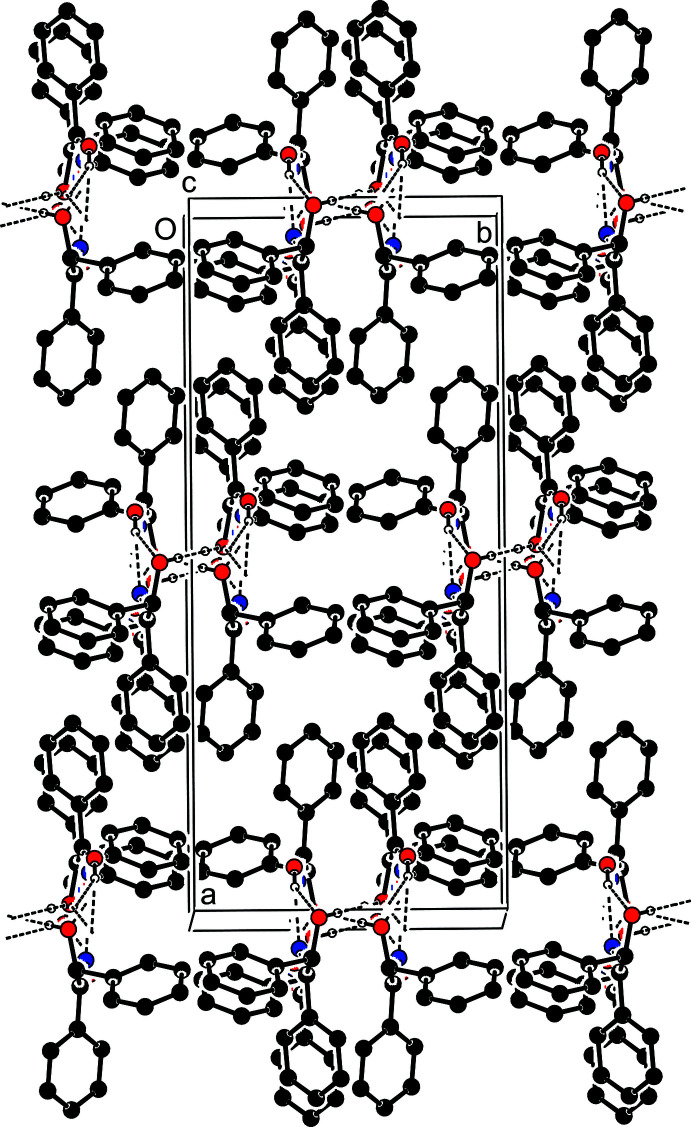
A packing diagram viewed down the *c* axis. The O—H_Oxm_⋯N_Oxm_, O—H_Hydr_⋯O_Hydr_, O—H′_Hydr_⋯O_Hydr_ and O—H_Oxm_⋯O_Hydr_ hydrogen bonds [Oxm = oxime and Hydr = hy­droxy] hydrogen bonds are shown as dashed lines. The remaining hydrogen atoms have been omitted for clarity.

**Table 1:** Hydrogen-bond geometry (Å, °) *Cg*2 is the centroid of the phenyl ring *B* (C9—C14).

*D*—H⋯*A*	*D*—H	H⋯*A*	*D*⋯*A*	*D*—H⋯*A*
O1—H1⋯O2^i^	0.88 (2)	2.47 (2)	3.2638 (19)	151 (1.47)
O1—H1⋯N1^i^	0.88 (2)	2.13 (2)	2.8236 (19)	135 (1.69)
O2—H2⋯O2^iii^	0.82 (4)	2.03 (2)	2.850 (3)	175 (1.99)
O2—H2′⋯O2^iv^	0.83 (4)	2.05 (3)	2.881 (3)	180 (2.38)
C4—H4⋯*Cg*2^vi^	0.93	2.91	3.792 (3)	159

Symmetry codes: (i) 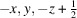; (iii) 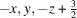; (iv) 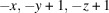; (vi) 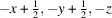.

**Table 2:** Selected interatomic distances (Å)

O1⋯C2	2.819 (3)	N1⋯H1^i^	2.13 (2)
O1⋯N1^i^	2.823 (2)	H2′⋯N1^iv^	2.83 (2)
C8⋯O1^ii^	3.381 (2)	H14⋯C6^ii^	2.79
O2⋯O2^iii^	2.850 (3)	C6⋯H8	2.66
O2⋯O2^iv^	2.881 (3)	C7⋯H10	2.92
O2⋯N1	2.577 (2)	C8⋯H2^iii^	2.76 (2)
O1⋯H2*A*	2.41	C8⋯H6	2.75
H8⋯O1^ii^	2.62	C8⋯H2′^iv^	2.87 (2)
O2⋯H10	2.87	C9⋯H2^iii^	2.92 (2)
O2⋯H1^i^	2.465 (17)	H1⋯H2′^i^	2.31
H2⋯O2^iii^	2.03 (2)	H2′⋯H1^ii^	2.59 (2)
H2′⋯O2^iv^	2.05 (3)	H2*A*⋯H13^v^	2.57
N1⋯N1^i^	2.867 (2)	H6⋯H8	2.12
N1⋯H2*A*	2.90	H8⋯H14	2.30
N1⋯H2′	2.49 (2)		

Symmetry codes: (i) 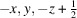; (ii) 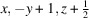; (iii) 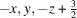; (iv) 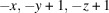; (v) 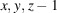.

**Table 3:** Experimental details

Crystal data	
Chemical formula	C_14_H_13_NO_2_
*M*_r_	227.25
Crystal system, space group	Monoclinic, *C*2/*c*
Temperature (K)	296
*a*, *b*, *c* (Å)	24.3559 (2), 10.7032 (2), 8.9667 (2)
β (°)	93.220 (2)
*V* (Å^3^)	2333.80 (7)
*Z*	8
Radiation type	Mo *K*α
μ (mm^−1^)	0.09
Crystal size (mm)	0.15 × 0.11 × 0.10

Data collection	
Diffractometer	Bruker APEXII QUAZAR three-circle
No. of measured, independent and observed [*I* > 2σ(*I*)] reflections	15784, 2673, 1781
*R*_int_	0.048
(sin θ/λ)_max_ (Å^−1^)	0.649

Refinement	
*R*[*F* ^2^ > 2σ(*F* ^2^)], *wR*(*F* ^2^), *S*	0.048, 0.124, 1.03
No. of reflections	2673
No. of parameters	158
H-atom treatment	H atoms treated by a mixture of independent and constrained refinement
Δρ_max_, Δρ_min_ (e Å^−3^)	0.17, −0.24

Computer programs: *APEX2* and *SAINT* (Bruker, 2012 ▸), *SHELXT* (Sheldrick, 2015*a* ▸), *SHELXL2018/3* (Sheldrick, 2015*b* ▸), *XP in *SHELXTL** (Sheldrick, 2008 ▸), *ORTEP-3 for Windows* and *WinGX* publication routines (Farrugia, 2012 ▸) and *PLATON* (Spek, 2020 ▸).

## supplementary crystallographic information

### Crystal data

**Table:**

C_14_H_13_NO_2_	*F*(000) = 960
*M**_r_* = 227.25	*D*_x_ = 1.294 Mg m^−^^3^
Monoclinic, *C*2/*c*	Mo *K*α radiation, λ = 0.71073 Å
*a* = 24.3559 (2) Å	Cell parameters from 2607 reflections
*b* = 10.7032 (2) Å	θ = 3.1–21.7°
*c* = 8.9667 (2) Å	µ = 0.09 mm^−^^1^
β = 93.220 (2)°	*T* = 296 K
*V* = 2333.80 (7) Å^3^	Block, colourless
*Z* = 8	0.15 × 0.11 × 0.10 mm

### Data collection

**Table:**

Bruker APEXII QUAZAR three-circle diffractometer	*R*_int_ = 0.048
Detector resolution: 8.3333 pixels mm^-1^	θ_max_ = 27.5°, θ_min_ = 1.7°
φ and ω scans	*h* = −31→31
15784 measured reflections	*k* = −13→13
2673 independent reflections	*l* = −11→11
1781 reflections with *I* > 2σ(*I*)	

### Refinement

**Table:**

Refinement on *F*^2^	0 restraints
Least-squares matrix: full	Hydrogen site location: inferred from neighbouring sites
*R*[*F*^2^ > 2σ(*F*^2^)] = 0.048	H atoms treated by a mixture of independent and constrained refinement
*wR*(*F*^2^) = 0.124	*w* = 1/[σ^2^(*F*_o_^2^) + (0.0438*P*)^2^ + 1.7468*P*] where *P* = (*F*_o_^2^ + 2*F*_c_^2^)/3
*S* = 1.03	(Δ/σ)_max_ < 0.001
2673 reflections	Δρ_max_ = 0.17 e Å^−^^3^
158 parameters	Δρ_min_ = −0.24 e Å^−^^3^

### Special details

**Table:**

Geometry. All esds (except the esd in the dihedral angle between two l.s. planes) are estimated using the full covariance matrix. The cell esds are taken into account individually in the estimation of esds in distances, angles and torsion angles; correlations between esds in cell parameters are only used when they are defined by crystal symmetry. An approximate (isotropic) treatment of cell esds is used for estimating esds involving l.s. planes.

### Fractional atomic coordinates and isotropic or equivalent isotropic displacement parameters (Å^2^)

**Table:**

	*x*	*y*	*z*	*U*_iso_*/*U*_eq_	Occ. (<1)
O1	0.06576 (6)	0.67667 (13)	0.19541 (13)	0.0449 (3)	
H1	0.0363 (8)	0.676 (2)	0.1336 (17)	0.067*	
O2	0.01347 (5)	0.60393 (13)	0.59737 (16)	0.0451 (4)	
H2	0.0037 (6)	0.602 (4)	0.684 (4)	0.068*	0.5
H2'	0.0059 (5)	0.544 (4)	0.542 (5)	0.068*	0.5
N1	0.04995 (6)	0.66307 (14)	0.34255 (15)	0.0373 (4)	
C1	0.14866 (7)	0.63331 (17)	0.41569 (19)	0.0362 (4)	
C2	0.17398 (8)	0.71174 (19)	0.3165 (2)	0.0463 (5)	
H2A	0.153310	0.771694	0.263426	0.056*	
C3	0.22965 (8)	0.7011 (2)	0.2964 (2)	0.0582 (6)	
H3	0.246113	0.754405	0.230145	0.070*	
C4	0.26080 (9)	0.6132 (3)	0.3727 (3)	0.0644 (7)	
H4	0.298150	0.605990	0.357871	0.077*	
C5	0.23633 (9)	0.5359 (2)	0.4713 (3)	0.0627 (6)	
H5	0.257333	0.475965	0.523500	0.075*	
C6	0.18100 (8)	0.5459 (2)	0.4939 (2)	0.0483 (5)	
H6	0.165180	0.493550	0.562321	0.058*	
C7	0.08910 (7)	0.64245 (15)	0.44001 (19)	0.0330 (4)	
C8	0.07095 (7)	0.62830 (17)	0.59889 (19)	0.0359 (4)	
H8	0.090620	0.557401	0.645918	0.043*	
C9	0.08441 (7)	0.74454 (18)	0.68979 (19)	0.0390 (4)	
C10	0.06246 (10)	0.8580 (2)	0.6490 (3)	0.0572 (6)	
H10	0.038643	0.864319	0.564624	0.069*	
C11	0.07566 (12)	0.9636 (2)	0.7335 (3)	0.0782 (8)	
H11	0.060924	1.040773	0.705199	0.094*	
C12	0.11041 (13)	0.9541 (3)	0.8583 (3)	0.0877 (10)	
H12	0.119636	1.025026	0.914105	0.105*	
C13	0.13143 (13)	0.8414 (3)	0.9010 (3)	0.0878 (10)	
H13	0.154334	0.834930	0.987246	0.105*	
C14	0.11882 (10)	0.7366 (2)	0.8165 (2)	0.0632 (6)	
H14	0.133716	0.659731	0.845480	0.076*	

### Atomic displacement parameters (Å^2^)

**Table:**

	*U*^11^	*U*^22^	*U*^33^	*U*^12^	*U*^13^	*U*^23^
O1	0.0426 (7)	0.0630 (9)	0.0294 (6)	−0.0031 (7)	0.0029 (5)	0.0015 (6)
O2	0.0370 (7)	0.0526 (8)	0.0470 (8)	−0.0134 (6)	0.0130 (6)	−0.0077 (6)
N1	0.0383 (8)	0.0432 (9)	0.0308 (7)	−0.0034 (7)	0.0054 (6)	−0.0022 (6)
C1	0.0342 (9)	0.0406 (10)	0.0338 (9)	−0.0037 (8)	0.0034 (7)	−0.0074 (7)
C2	0.0434 (11)	0.0530 (12)	0.0429 (11)	−0.0069 (9)	0.0069 (8)	−0.0014 (9)
C3	0.0432 (12)	0.0805 (16)	0.0521 (12)	−0.0137 (11)	0.0145 (10)	−0.0048 (11)
C4	0.0346 (11)	0.0986 (19)	0.0609 (14)	0.0020 (12)	0.0093 (10)	−0.0156 (13)
C5	0.0434 (13)	0.0808 (17)	0.0636 (14)	0.0150 (11)	−0.0004 (11)	−0.0021 (12)
C6	0.0399 (11)	0.0557 (12)	0.0495 (11)	0.0024 (9)	0.0044 (9)	0.0001 (9)
C7	0.0340 (9)	0.0317 (9)	0.0335 (9)	−0.0053 (7)	0.0037 (7)	−0.0035 (7)
C8	0.0305 (9)	0.0408 (10)	0.0367 (9)	−0.0052 (7)	0.0049 (7)	0.0021 (8)
C9	0.0397 (10)	0.0472 (11)	0.0309 (9)	−0.0124 (8)	0.0087 (7)	−0.0013 (8)
C10	0.0651 (14)	0.0509 (13)	0.0559 (13)	−0.0012 (11)	0.0056 (11)	−0.0080 (10)
C11	0.093 (2)	0.0522 (15)	0.092 (2)	−0.0077 (13)	0.0314 (17)	−0.0185 (14)
C12	0.114 (2)	0.093 (2)	0.0601 (16)	−0.0545 (19)	0.0367 (16)	−0.0357 (16)
C13	0.108 (2)	0.112 (3)	0.0429 (14)	−0.059 (2)	−0.0020 (14)	−0.0092 (15)
C14	0.0712 (15)	0.0747 (16)	0.0428 (11)	−0.0246 (13)	−0.0062 (10)	0.0051 (11)

### Geometric parameters (Å, º)

**Table:**

O1—N1	1.4026 (18)	C5—H5	0.9300
O1—H1	0.88 (2)	C6—H6	0.9300
O2—C8	1.424 (2)	C7—C8	1.523 (2)
O2—H2	0.82 (4)	C8—C9	1.513 (2)
O2—H2'	0.83 (4)	C8—H8	0.9800
N1—C7	1.276 (2)	C9—C10	1.368 (3)
C1—C6	1.388 (3)	C9—C14	1.376 (3)
C1—C2	1.392 (3)	C10—C11	1.388 (3)
C1—C7	1.482 (2)	C10—H10	0.9300
C2—C3	1.383 (3)	C11—C12	1.369 (4)
C2—H2A	0.9300	C11—H11	0.9300
C3—C4	1.368 (3)	C12—C13	1.356 (4)
C3—H3	0.9300	C12—H12	0.9300
C4—C5	1.372 (3)	C13—C14	1.379 (4)
C4—H4	0.9300	C13—H13	0.9300
C5—C6	1.378 (3)	C14—H14	0.9300
			
O1···C2	2.819 (3)	N1···H1^i^	2.13 (2)
O1···N1^i^	2.823 (2)	H2'···N1^iv^	2.83 (2)
C8···O1^ii^	3.381 (2)	H14···C6^ii^	2.79
O2···O2^iii^	2.850 (3)	C6···H8	2.66
O2···O2^iv^	2.881 (3)	C7···H10	2.92
O2···N1	2.577 (2)	C8···H2^iii^	2.76 (2)
O1···H2A	2.41	C8···H6	2.75
H8···O1^ii^	2.62	C8···H2'^iv^	2.87 (2)
O2···H10	2.87	C9···H2^iii^	2.92 (2)
O2···H1^i^	2.465 (17)	H1···H2'^i^	2.31
H2···O2^iii^	2.03 (2)	H2'···H1^ii^	2.59 (2)
H2'···O2^iv^	2.05 (3)	H2A···H13^v^	2.57
N1···N1^i^	2.867 (2)	H6···H8	2.12
N1···H2A	2.90	H8···H14	2.30
N1···H2'	2.49 (2)		
			
N1—O1—H1	109.5	O2—C8—C9	109.82 (14)
C8—O2—H2	109.5	O2—C8—C7	110.26 (14)
C8—O2—H2'	109.5	C9—C8—C7	110.90 (14)
C7—N1—O1	115.36 (14)	O2—C8—H8	108.6
C6—C1—C2	118.06 (17)	C9—C8—H8	108.6
C6—C1—C7	120.16 (16)	C7—C8—H8	108.6
C2—C1—C7	121.77 (17)	C10—C9—C14	119.1 (2)
C3—C2—C1	120.5 (2)	C10—C9—C8	121.08 (17)
C3—C2—H2A	119.8	C14—C9—C8	119.82 (19)
C1—C2—H2A	119.8	C9—C10—C11	120.1 (2)
C4—C3—C2	120.8 (2)	C9—C10—H10	119.9
C4—C3—H3	119.6	C11—C10—H10	119.9
C2—C3—H3	119.6	C12—C11—C10	119.9 (3)
C3—C4—C5	119.3 (2)	C12—C11—H11	120.0
C3—C4—H4	120.4	C10—C11—H11	120.0
C5—C4—H4	120.4	C13—C12—C11	120.2 (2)
C4—C5—C6	120.8 (2)	C13—C12—H12	119.9
C4—C5—H5	119.6	C11—C12—H12	119.9
C6—C5—H5	119.6	C12—C13—C14	119.9 (3)
C5—C6—C1	120.7 (2)	C12—C13—H13	120.0
C5—C6—H6	119.7	C14—C13—H13	120.0
C1—C6—H6	119.7	C9—C14—C13	120.7 (3)
N1—C7—C1	127.62 (15)	C9—C14—H14	119.7
N1—C7—C8	114.40 (15)	C13—C14—H14	119.7
C1—C7—C8	117.97 (15)		
			
C6—C1—C2—C3	0.6 (3)	C1—C7—C8—O2	−163.47 (15)
C7—C1—C2—C3	179.89 (18)	N1—C7—C8—C9	−104.31 (17)
C1—C2—C3—C4	0.3 (3)	C1—C7—C8—C9	74.7 (2)
C2—C3—C4—C5	−0.7 (3)	O2—C8—C9—C10	−61.5 (2)
C3—C4—C5—C6	0.0 (4)	C7—C8—C9—C10	60.6 (2)
C4—C5—C6—C1	1.0 (3)	O2—C8—C9—C14	117.82 (19)
C2—C1—C6—C5	−1.3 (3)	C7—C8—C9—C14	−120.07 (19)
C7—C1—C6—C5	179.46 (18)	C14—C9—C10—C11	1.1 (3)
O1—N1—C7—C1	1.0 (3)	C8—C9—C10—C11	−179.54 (19)
O1—N1—C7—C8	179.88 (13)	C9—C10—C11—C12	−0.5 (4)
C6—C1—C7—N1	−141.68 (19)	C10—C11—C12—C13	−0.9 (4)
C2—C1—C7—N1	39.1 (3)	C11—C12—C13—C14	1.5 (4)
C6—C1—C7—C8	39.5 (2)	C10—C9—C14—C13	−0.5 (3)
C2—C1—C7—C8	−139.75 (17)	C8—C9—C14—C13	−179.8 (2)
N1—C7—C8—O2	17.5 (2)	C12—C13—C14—C9	−0.9 (4)

Symmetry codes: (i) −*x*, *y*, −*z*+1/2; (ii) *x*, −*y*+1, *z*+1/2; (iii) −*x*, *y*, −*z*+3/2; (iv) −*x*, −*y*+1, −*z*+1; (v) *x*, *y*, *z*−1.

### Hydrogen-bond geometry (Å, º)

Cg2 is the centroid of the phenyl ring *B* (C9—C14).

**Table:**

*D*—H···*A*	*D*—H	H···*A*	*D*···*A*	*D*—H···*A*
O1—H1···O2^i^	0.88 (2)	2.47 (2)	3.2638 (19)	151 (1.47)
O1—H1···N1^i^	0.88 (2)	2.13 (2)	2.8236 (19)	135 (1.69)
O2—H2···O2^iii^	0.82 (4)	2.03 (2)	2.850 (3)	175 (1.99)
O2—H2′···O2^iv^	0.83 (4)	2.05 (3)	2.881 (3)	180 (2.38)
C4—H4···*Cg*2^vi^	0.93	2.91	3.792 (3)	159

Symmetry codes: (i) −*x*, *y*, −*z*+1/2; (iii) −*x*, *y*, −*z*+3/2; (iv) −*x*, −*y*+1, −*z*+1; (vi) −*x*+1/2, −*y*+1/2, −*z*.
